# Pregnancies following Protocols for Repetitive Synchronization of Ovulation in Primiparous Buffaloes in Different Seasons

**DOI:** 10.3390/vetsci9110616

**Published:** 2022-11-07

**Authors:** Giorgio A. Presicce, Domenico Vistocco, Massimo Capuano, Luigi Navas, Angela Salzano, Giovanna Bifulco, Giuseppe Campanile, Gianluca Neglia

**Affiliations:** 1 Agenzia Regionale per lo Sviluppo e l’Innovazione dell’Agricoltura del Lazio (ARSIAL), 00162 Rome, Italy; 2 Department of Political Science, University of Naples Federico II, 80131 Naples, Italy; 3 Freelance Veterinarian, 71100 Foggia, Italy; 4 Department of Veterinary Medicine and Animal Productions, University of Naples Federico II, 80137 Naples, Italy

**Keywords:** artificial insemination, P_4_, Ovsynch, buffalo species, synchronization protocols, primiparous, pregnancy rate, reproductive efficiency

## Abstract

**Simple Summary:**

Artificial Insemination (AI) is mainly used after estrus synchronization in buffalo, and consecutive synchronization protocols are used to enhance reproductive efficiency. In this study, two different synchronization protocols have been used: Ovsynch vs. a P_4_-administration, and their efficiency in primiparous animals has been evaluated in different seasons for up to four cycles of re-synchronization protocols. Results show that the pregnancy rate upon the initial AI tends to be higher in P_4_ treated buffaloes, and that AI efficiency after re-synchronization through P_4_ is higher than the Ovsynch protocol. In conclusion, synchronization treatments must be selected according to the season of the year. The results derived from this study could be useful for buffalo breeders who want to improve the reproductive efficiency in primiparous animals in commercially managed buffalo herds.

**Abstract:**

Primiparous buffaloes were tested in two periods of the year characterized, by either low or high reproductive efficiency. They were subjected to two protocols for synchronization of ovulation: (i) Ovsynch (OV) and (ii) progesterone based (P_4_) treatment. After calving, the animals underwent a series of four cycles of re-synchronization protocols. The season did not affect pregnancy rates when the results of the two treatments were pooled together with regard to the first synchronization protocol, followed by AI. Pregnancy rates were similar during the low breeding season (50.3% vs. 57.4% in OV and P_4_, respectively), but different during the high breeding season (50.4% vs. 67.7% in OV and P_4_, respectively; *p* = 0.000). Logistic regression confirmed a significant effect of treatment and season interaction on pregnancy (*p* = 0.003). Following re-synchronization, a treatment by season interaction was detected during the low breeding season (odds ratio = 2.233), in favor of P_4_. Finally, a survival analysis showed a better response of animals subjected to P_4_ treatment from the second AI onward. In conclusion, the pooled data of pregnancy rates from both treatments between seasons are not different following AIs. Better results, though, were obtained from the implementation of P_4_ treatment, and are recorded in a season-fashioned mode when the comparison is made following first or cumulative AIs.

## 1. Introduction

Nowadays, reproductive management in buffaloes is more and more reliant on the implementation of technologies that were previously adopted only in cattle [1,2,3]. From the use of pedometers to the adoption of AI, coupled with the refinement of existing protocols for the synchronization of ovulation [4], reproductive efficiency has witnessed a significant improvement compared to prior years [5], especially when such strategies are exploited during the course of unfavorable seasons [6,7,8]. In fact, when considering reproductive efficiency, buffaloes are animals characterized by a tendency to perform better during the period of the year when light hours are decreasing, or in autumn and winter months, when the number of night hours is higher than light hours [9,10]. In this respect, a management strategy that eliminates from the “good reproductive season” the possibility for buffaloes to naturally mate has been devised and implemented. The reason behind this strategy derives and has taken root in the realm of social habits and market demands, which are higher during the spring-summer period of the year (March to August), and has been implemented for the last twenty years in countries like Italy. This is accomplished by removing bulls from the herds (or interrupting the application of AI) at the beginning of autumn and reintroducing them (or re-starting with AI) in spring, thus allowing a higher rate of pregnancies and calving coinciding with the period of the year where the demands for buffalo milk and milk-derived products are higher: a strategy which has been termed Out of Breeding Season Mating technique (OBSM) [9,10].

For this strategy to be effective, though, the negative influence of months characterized by longer daylight hours on reproductive performance must be counteracted, and for this to be achieved, the use of AI is imperative, together with the selection of the right females to enter protocols for the synchronization of ovulation [4,10]. The application of AI allows breeders to achieve a proper distribution of calving throughout the year [4], and to reduce the phenomenon of anoestrus that both males and females encounter during periods of increasing daylight length [1,4,6]. Furthermore, breeders must also consider an intrinsic differential sensitivity existing between heifers, primiparous, and pluriparous animals, with regard to the effect of light hours on their reproductive function, with heifers being less affected by the negative effects of long daylight hours [11,12]. Protocols for the synchronization of ovulation during these days are by far considered the best fitted approach in buffaloes, in opposition to protocols for the synchronization of estrus. Two of them are taken into consideration in this study: the first, Ovsynch, developed by Pursley et al. [13], can be adopted in cycling animals characterized by follicular growth up to the size for follicle ovulation to occur, and subsequent CL development and function [14]. The second and alternative approach used in this study is based on P_4_ administration, and can be adopted especially on animals which are either non-cycling, or showing unclear signs of cyclic activity [6,11]. In this case, hormonal administration has to be considered through the use of an internal P_4_ releasing device together with either FSH or PMSG to foster and sustain follicle development [15,16,17]. It is clear that these treatments differ substantially in their hormonal mechanism for the synchronization of wave development and ovulation, but their use can lead to a successful AI. Therefore, under such assumptions and with the possibility to implement both protocols according to the physiological status of the animals, even the unfavorable season can be successfully used to perform AI on buffaloes. 

In a previous study carried out on buffalo heifers [16], it was shown that it is possible to envision specific protocols for the synchronization and re-synchronization of ovulation up to six times, through the implementation of the two different schemes of hormonal administration. In that study, the response to both treatments was similar with satisfactory synchronization and pregnancy rates. It is important to emphasize that all treated animals were selected for being in a cycling condition, although in a period of the year usually characterized by low reproductive efficiency. That research has clearly evidenced that most of the pregnancies were obtained within the first three synchronization treatments, and that P_4_-based protocol was overall more effective for the establishment of pregnancies. More specifically, P_4_-based protocol significantly increased the possibility to determine pregnancy in subsequent synchronization following the first one [16]. Similarly, both treatments in pluriparous buffaloes have been proven effective in obtaining acceptable to good synchronization and pregnancy rates in commercially managed herds [18], reducing also the incidence of embryonic mortality [19]. A number of strategies and variables come into play when trying to improve the efficiency derived from the application of the two treatments in pluriparous buffaloes, such as the availability of a large follicle at the time of first GnRH administration in the course of the Ovsynch treatment [20]. Therefore, it is reasonable that the physiological status of the animals may affect the response to different synchronization treatments and, consequently, pregnancy outcome following AI in buffalo.

As a follow-up investigation, in this study the same two protocols for the synchronization of estrus in primiparous animals in a farm in the South of Italy, from March to December of the same year, were compared. The animals were synchronized for a total number of four times in order to: (i) record the overall fertility and established pregnancies across the entire period under study, and (ii) verify the effect of each protocol on the establishment of pregnancy into the two distinct seasons: March to July (increasing daylight hours IDH— out of breeding season) and August to December (decreasing daylight hours DDH—breeding season). 

## 2. Materials and Methods

### 2.1. Farm Management and Animals 

The study and all standard veterinary procedures received institutional approval by the Ethical Animal Care and Use Committee of the University of Naples Federico II (Protocol No. PG/2021/0130478 del 16 December 2021). The study was carried out in a commercial farm located in Cerignola (FG) in the South of Italy at 41.1° N latitude and 15.5° E longitude, on a total of 1010 primiparous buffaloes. Approximately 3500 buffaloes were bred at that time in the farm and maintained in open yards allowing 15 m^2^/head. Adult lactating buffaloes were milked twice daily and received, constantly throughout the year, a total mixed ration consisting of 55% forage and 45% concentrate. The diet was characterized by 0.92 milk forage units⁄kg of dry matter, 15% crude protein⁄dry matter, 40% neutral deters fiber and 20% starch in a group pen situation. 

The reproductive management of the farm was carried out by using artificial insemination (AI) following synchronization of ovulation (see below), according to schedules already previously described and adopted [16,18]. Briefly, all lactating adult buffaloes underwent clinical examination after calving to evaluate the conditions of the genital tract: only animals in good health and without any abnormalities of the uterus or ovaries were included in the study. Furthermore, the cyclic status of the animals was evaluated by two clinical and ultrasound examinations 12 days apart: only animals that showed the presence of a corpus luteum in at least one examination were recruited to enter protocols for synchronization of ovulation and AI. If a corpus luteum was not detected, the animals underwent further examinations and were assigned to the synchronization protocols only when the cyclic status was confirmed. When the animals were recruited the Body Condition Score (BCS) of each subject was assessed by using the 1 to 9 scale [21]. Simultaneously, ambient temperature and relative humidity (AT and RH, respectively) were recorded throughout the study (from March to December) by using a weather station located approximately 3 km from the farm. These data were used to calculate the Temperature-Humidity Index (THI), according to the following equation [22]:

THI = (1.8 × AT + 32) − (0.55 − 0.55 × RH) × (1.8 × AT + 32) − 58

where AT is expressed in degrees Celsius and RH as a fraction of the unit. The (1.8 × AT + 32) term is used for the conversion from degree Celsius to Fahrenheit.

### 2.2. Experimental Design: First Synchronization Treatments, TAI and Ultrasound

After selection of animals, all primiparous buffaloes were randomly assigned to receive one of the two synchronization protocols:

1. Group OV underwent an Ovsynch-TAI program [14]. Briefly, a GnRH analogous (buserelin acetate, 12 mg; Receptal^®^, Intervet, Milan, Italy) was administered on day 0, followed by a PGF_2α_ (dinoprost; Dinolytic^®^, Zoetis, Milan, Italy) on day 7 and a second GnRH administration on Day 9. Timed AI was carried out on day 10 at 60 h and 16 h after PGF_2α_ and the last GnRH administration.
2. Group P_4_ was treated by a progesterone (P_4_) based protocol [16]. Briefly, the animals received a GnRH analogous (buserelin acetate, 12 mg; Receptal^®^, Intervet, Milan, Italy) administration on Day 0, together with the insertion of aP_4_-based intravaginal device (CIDR^®^, Zoetis, Milan, Italy), that was maintained for 10 days. On the day of device removal (Day 10), both 750 IU of equine chorionic gonadotrophin (eCG; Folligon^®^, Intervet MSD, Milan, Italy) and 25 mg of dinoprost (Dinolytic^®^, Zoetis, Milan, Italy) were administered, followed two days later (Day 12) by an administration of GnRH analogous (buserelin acetate, 12 mg; Receptal^®^, Intervet, Milan, Italy). Timed AI was also performed in this case 60 h and 16 h after PGF_2α_ and GnRH administration, respectively.

On the day of TAI, an ultrasound examination of the ovaries was carried out on all buffaloes by using a portable Sonoace Pico (Medison, Seoul, Korea) equipped with a 10 MHz linear transducer for transrectal examination in large animals: only buffaloes with a follicle higher than 1 cm with a tonic uterus were inseminated. Timed AIs were performed by the same experienced veterinarian by using frozen/thawed semen of 10 bulls of proven fertility. All buffaloes with a follicle higher than 1 cm and tonic uterus underwent AI, independently from other signs of estrus. Pregnancy diagnosis was performed on day 27 by ultrasound. 

### 2.3. Experimental Design: Re-Synchronization

**Figure 1:**
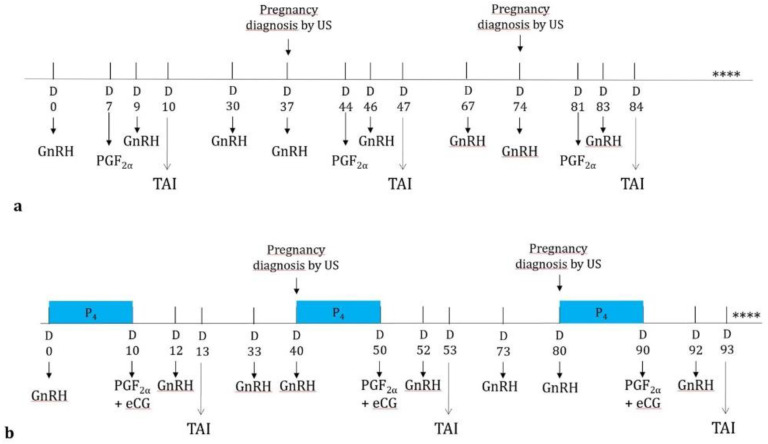
Ovsynch (**a**) and progesterone based (**b**) protocols for re-synchronization of ovulation in primiparous buffaloes. (**** this schedule was repeated for a maximum of 4 times).

Throughout the study, the efficiency of the two synchronization protocols followed by the first AI, and subsequent re-synchronization protocols and AIs in a timely fashion way [16,23], were tested in primiparous buffaloes. All animals, independently of the treatment, received an additional GnRH administration 20 days after AI to support a possible pregnancy, thus reducing embryonic mortality [19]. Seven days later, the animals underwent ultrasound examination to assess the presence of a pregnancy, and only buffaloes that were not detected pregnant were assigned to receive a re-synchronization protocol. In detail, non-pregnant animals in group OV were administered GnRH on the same day of ultrasound pregnancy check, PGF2α seven days later and again GnRH after two days, followed finally by timed AI the next day (Figure 1). Similarly, animals in Group P_4_ received a GnRH administration together with the insertion of a P_4_ based intravaginal device for 10 days and at removal, 750 IU of equine chorionic gonadotrophin and 25 mg of dinoprost. An additional GnRH was administered two days later, and TAI was performed 16 h after GnRH (Figure 1). This schedule was repeated a maximum of 4 times, although, for commercial reasons, some buffaloes were excluded from the trial after the first TAI and assigned to be naturally mated. Both treatments were carried out throughout the year, in order to compare the efficiency of re-synchronization treatments during the IDH and DDH. Finally, some animals were inseminated for the first time during the transition period (January–February) and, therefore, were not included in the trial for evaluating the pregnancy rate after the first AI. However, since they received the second, third and fourth AI during IDH and DDH were considered during the resynchronization.

### 2.4. Statistical Analysis

The study was approached from a dual perspective: the pregnancy rate at the first AI was investigated using a logistic regression model [24] while the time to become pregnant with subsequent AIs was analyzed through a survival analysis [25]. A Cox regression model [26] allowed us to study the effect of covariates on the time to become pregnant. Such an analysis strategy focused on the factors influencing the success at the first AI and at subsequent AIs. In particular, the comparison between the two considered synchronization protocols in terms of their efficiency was carried out by taking into account the effect of the age of animals, interval from parturition to first AI, and season in which the insemination took place, distinguishing high breeding and low breeding season.

## 3. Results

### 3.1. Farm Management and Animals

No differences were recorded in terms of the milk yield of the animals during the trial (10.2 ± 0.6 vs. 10.4 ± 0.5 kg/day respectively in OV and P_4_ treatment). The BCS of the enrolled buffaloes was similar between the two experimental groups (7.3 ± 0.2 vs. 7.4 ± 0.3 respectively in OV and P_4_ treatment). The THI values ranged from 64.4 ± 5.7 in the IDH season to 66.3 ± 6.7 in the DDH season, *p* > 0.05.

### 3.2. Synchronization Protocols and Pregnancies following the First AI

**Figure 2:**
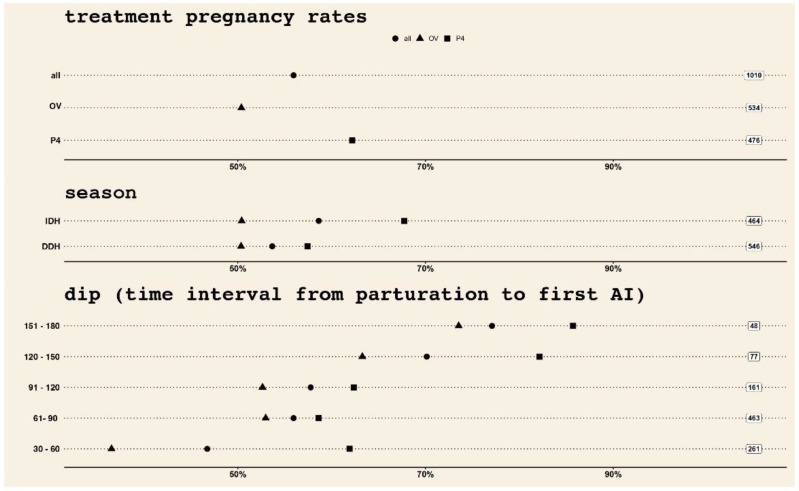
From top to bottom: overall pregnancy rate (circle), OV (triangle) and progesterone (square) pregnancy rates with regard to (i) treatment (top panel), (ii) season in which the insemination took place (middle panel), and (iii) dip, interval from parturition to first AI (bottom panel). Pregnancy rates refer only to the first AI after parturition.

Whenever the two treatments for the synchronization of ovulation will be compared below, the first of the two sets of data will always refer to the OV protocol. Figure 2 offers a graphical summary of pregnancy rates at first AI, in terms of synchronization protocol, season in which the insemination took place and interval from parturition to first AI (three panels of the graph, from the top). In each panel, the overall pregnancy rate is depicted with a circle, while the pregnancy rates corresponding to each treatment using a triangle for OV and a square for P_4_.

The following results emerge when exploiting Pearson’s Chi-squared test with Yates’ continuity correction: similar pregnancy rates when the two seasons were compared (from a total of 1010 animals subjected to the first AI, 53,7% vs. 58.6% were recorded pregnant during IDH and DDH, respectively, *p* = 0.1). Likewise, when the two treatments were compared within the IDH season, similar pregnancy rates were confirmed (50.3% vs. 57.4%; *p* = 0.1). On the contrary, within DDH season, pregnancy rates differed between the two treatments (50.4% vs. 67.7%; *p* = 0.000). See Table 1 for a cumulative and concise descriptive summary.

The time encompassing from parturition to the first AI is another important point to be considered when confronting seasons and treatments for the assessment of synchronization of ovulation and the establishment of pregnancies in primiparous buffaloes. In that regard, Figure 2 clearly shows a better effect of P_4_-based treatment for the synchronization of ovulation in primiparous buffaloes, not only in terms of overall pregnancy rates, but also when the same are confronted between the seasons considered in this study, and finally when the two treatments are compared when considering the distance of first AI from parturition.

To explain the reasons of success of the first AI following parturition, a logistic regression model was implemented by including several regressors such as treatment, season, time interval from parturition to first AI (dip), age at parturition and season by treatment interaction. It appears that the age of the animals and the treatment for synchronization of ovulation do not significantly contribute to the model, whereas dip and season by treatment interaction positively affect the model, although the first of these last two variables is characterized by a very low coefficient (0.01). A higher coefficient (0.8) with a statistical significance of *p* = 0.003 is instead associated to the interaction term. The coefficient of each regressor can be put in terms of effect on the odds ratio. Detailed information (odds ratio, sign of the coefficient and corresponding *p*-value) is given in Table 2.

**Figure 3:**
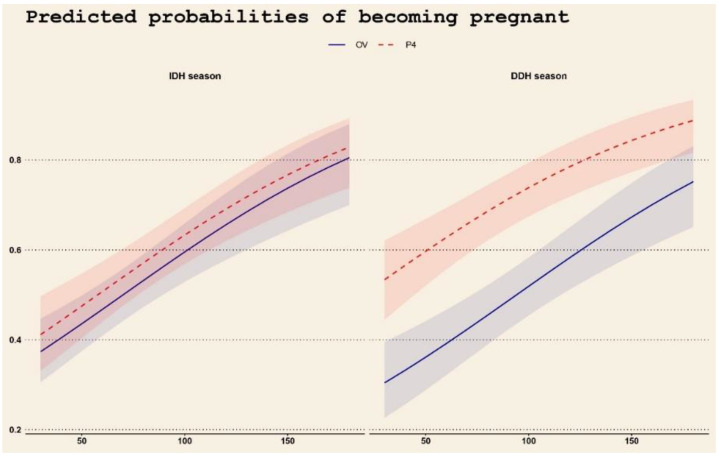
Marginal effects of dip regressor (horizontal axis) over seasons (panels) and treatments (lines) on the predicted probabilities of becoming pregnant in primiparous buffaloes synchronized by Ovsynch (OV) or Progesterone based treatment (P_4_) that underwent AI during periods characterized by Increasing Daylight Hours (IDH) and Decreasing Daylight Hours (DDH).

A closer look at the interdependence between treatment, season, and time interval from parturition to first AI reveals that while the regressor treatment alone does not significantly affect the expected result (pregnancy), its interaction with season shows a significant difference between treatments in the DDH season. This is well evidenced in Figure 3, depicting the marginal effects of dip on the response variable, considering treatment and seasons. In particular, the values of dip are depicted on the horizontal axis, while the vertical axis reports the predicted probabilities of becoming pregnant. The two panels refer to the season, IDH on the left and DDH on the right, and the two lines to the treatment, being solid red line for OV and dashed blue line for P_4_. The shaded areas around the lines show the 95% confidence intervals for the predicted probabilities.

### 3.3. Synchronization Protocols and Pregnancies following Consecutive AIs

To the end of exploring the efficiency of re-synchronization protocols on pregnancy rates following the first AI, only animals for which the first insemination was unsuccessful were analyzed. A maximum number of four AIs was allowed to this goal, even if there are animals that never became pregnant during the observed period. Descriptive statistics such as Pearson’s Chi-squared test with Yates’ continuity correction is reported in Table 3.

**Figure 4:**
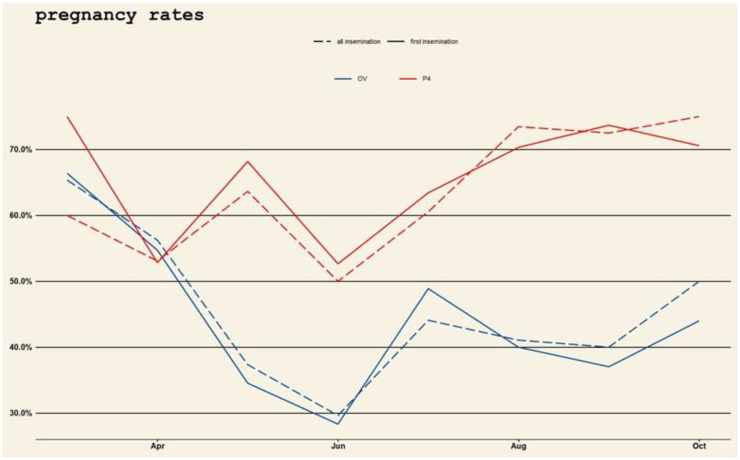
Descriptive analysis and tendency of pregnancies over months (horizontal lines), comparing the two treatments (dashed lines for Ovsynch (OV) and dotted lines for Progesterone based treatment (P_4_)) and the rates at first Artificial Insemination (dotted red lines) and subsequent inseminations (dashed lines for OV and dotted lines for P_4_).

A significantly (*p* < 0.01) higher pregnancy rate was recorded in buffaloes re-synchronized by P_4_-based protocol, compared with the OV counterparts only during IDH period. By analyzing season and treatment over time, P_4_ based treatment shows a clear superior pattern over OV. This is summarized in Figure 4 where the pattern of pregnancy rates over time is depicted in the horizontal axis, and where data related to the first AI, data related to subsequent AIs (dotted red lines) and data related to the two treatments (dashed lines for OV and dotted lines for P_4_), are detailed. 

**Figure 5:**
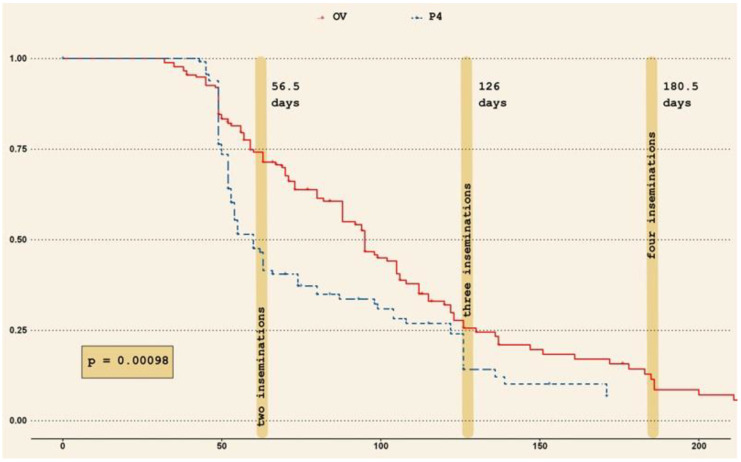
Survival curves showing the effect of treatments and interval from parturition on pregnancy rates, following consecutive re-synchronization protocols after the first artificial insemination (AI) in primiparous buffaloes. Solid line refers to Ovsynch (OV) and dashed curve to Progesterone based treatment (P_4_) (*p* = 0.00098). The vertical stripes depict the median number of days to become pregnant at the second, third and fourth AI.

A survival analysis on the animals requiring more than one AI to become pregnant allowed us to investigate the effect of season and treatment over the time needed to become pregnant. Figure 5 depicts the survival curves for the two treatments (solid curve refers to OV and dashed curve to P_4_). The median number of days to become pregnant at the second, third, and fourth AI are also shown on the plot. Once again, the chances of getting primiparous buffaloes pregnant from the second AI onward are higher when P_4_ treatment is implemented on animals. The same results are confirmed also through a Cox regression model (data not shown).

## 4. Discussion

In a timespan of two decades, the use of AI for the control of reproductive efficiency in buffaloes has gained momentum, thanks to mushrooming literature made available on the different strategies to synchronize estrus and ovulation [3,4,12,17,23]. In addition, the adoption and common use of AI in buffaloes in recent years has benefited from earlier studies that have highlighted the physiological mechanisms and hormonal patterns of wave follicular dynamics [27,28]. A substantial impediment to the successful application of this technology for genetic improvement in the buffalo species emerged when, at the beginning, AI was linked to the synchronization of estrus. In fact, due to buffalo’s inherent physiological characteristics [11,29,30], AI on natural or induced estrus did not provide satisfying results [30]. Only with the advent and improvement of protocols for the synchronization of ovulation did AI find the favor of breeders, thanks to more acceptable and consistent pregnancy rates [4]. 

It must be highlighted that a large variability of results over the years has been obtained, following the application of the two protocol object of this study (OV-based vs. P_4_-based treatment). Such variability is due mostly to the season of reference when the study was carried out, parity of animals, farm management, buffalo subspecies and breed, country of origin, and finally to slight modifications made to the treatments themselves [12,16,23,31,32,33].

Despite earlier trials on the use of OV-based protocol in primiparous buffaloes, to our knowledge, this is the first study comparing the two most important protocols for synchronization of ovulation in this category. The importance of parity on reproductive performance in large ruminants is widely acknowledged [34,35]. Some authors [36,37] recorded lower success in the first insemination in first-parity dairy cows, probably for the severe negative energy balance (NEB) of these animals in the post-partum period [38]. Likewise, it cannot be excluded that primiparous buffaloes are particularly sensitive to environmental conditions and suffer from a longer inter-calving period due mostly to a prolonged NEB after delivery. This phenomenon is caused and sustained by the temporal parallel subsistence of requirements for final somatic growth and milk production [38]. Buffaloes are animals that need constant support to exploit their reproductive efficiency, and this is where the adoption of AI, together with the two protocols for the synchronization of ovulation, come to the rescue. In this regard, there have not yet been studies and results available on the efficiency of AI following the two synchronization protocols performed on the two reproductive seasons, except for those carried out in Italy by our research group on heifers [14]. It is worth highlighting that in this study, the two protocols were repeated up to four times and at different intervals from calving. 

From this study, it emerges that when the results from the two treatments are pooled, no difference in pregnancy rate can be found between the two seasons. A difference between these treatments, though, can be found in the high reproductive season following the first AI, and in the low reproductive season when all four AIs are considered, with the P_4_ based treatment in both cases favoring a significative higher pregnancy rate. A possible explanation for an overall similar good pregnancy rate, recorded following both treatments across seasons and months considered, can be found in the starting cycling condition in all animals enrolled in the study [39,40]. In fact, while the Ovsynch-TAI program is particularly effective in cyclic subjects [41], P_4_ based treatments result in high pregnancy rate also in non cyclic animals, in both cattle [42] and buffalo increase [4]. The reasons for this can be found in the physiological mechanisms of action: the Ovsynch-TAI can induce ovulation only if a follicle that already reached the deviation stage is present on the ovary [43]. On the contrary, P_4_ administered during P_4_ based treatments synchronization protocols is able to support follicles growth regulating gonadotrophin pulses also acting at pituitary level, with an effective impact on the follicular wave growth magnitude and speed [11,44]. However, it is known that buffaloes are considered “tendentially” seasonal animals, since their reproductive activity increases during periods of decreasing daylight length, but is reduced when daylight hours increase [11]. This means the possibility of recording and identifying cyclic subjects during periods of daylight increases. Nevertheless, under some conditions, a prevalence of effect on pregnancy rate derived from the P_4_-based treatment can be envisaged by the possibility that such treatment may in fact fit better or better rescue those animals in need of a higher support given by P_4_ itself and hormones supporting follicle growth and function such as FSH or PMSG [45].

Similarly to heifers [16], in this study most pregnancies in primiparous buffaloes were obtained within the first three AIs. It is worth noting that in the class of animals grouped between 30 to 60 days from calving and subjected to the first AI, P_4_-based treatment definitely gave better pregnancy rates. This is the period closest to the first calving, when buffaloes exit from the time needed for uterine involution [30] and enter into resumption of cyclicity; a time where both the administration of P_4_ and either FSH or PMSG can give full and better support for follicle development, leading to a successful ovulation [46]. This is substantiated by the data recorded in the group of animals farther away from calving, over 60 to 90 days, for which the type of treatment does not significantly affect pregnancy rates. In addition, logistic regression analysis showed that distance from calving and treatment by season interaction mostly affect the ratio of obtaining pregnancies. Although not very high, the first of the two odds are in line with physiological consistent arguments that see an animal becoming pregnant within a time frame from calving that takes into consideration the two elements above noted, uterine involution and resumption of cyclicity. The high season and treatment interaction confirm a superior efficacy of the P_4_-based treatment on primiparous buffaloes undergoing their first AI.

When considering the total of AIs to which animals were subjected, the comparison of treatments and their interactions with the variables considered in this study confirmed again the higher efficiency of P_4_-based over OV-based treatment across the two seasons and the single months encompassing the period of study under scrutiny. Finally, the superiority of P_4_-based over OV-based treatment, when all resynchronization treatments and AIs were considered, has been confirmed also by the Cox regression model, which revealed a faster probability of achieving pregnancy over time.

Although some differences emerged between seasons, treatments, and their interaction, the present study underlies a good efficacy of results in terms of pregnancy rates when the two protocols for synchronization of ovulation are applied to cycling primiparous buffaloes. It was already highlighted that, among all the criteria for selecting buffaloes, one of the newest refers to the sensitivity to environmental conditions, in particular light [11]. It is known that heifers suffer less when compared to pluriparous buffaloes, from sensitivity to the light stimulus, but even among adult buffaloes some animals may be less sensitive than others. Therefore, by keeping attention in selecting more and more buffaloes which are less sensitive to environmental conditions, it is possible that in the future, the majority of buffaloes will be found cycling. This process can definitely be accelerated through the utilization of new technologies such as genomics, accomplished with AI utilization, as it is performed in cattle [47]. The difference in results from the application of treatments for the synchronization of ovulation would become negligible up to a point where only a small portion of animals will be in need of specific treatments such as those requiring the administration of P_4_ and either FSH or PMSG.

## 5. Conclusions

Considering the high economic value of buffalo milk in Italy for producing mozzarella di bufala Campana cheese PDO, the management of reproduction is particularly important, and the utilization of hormonal treatments may represent a cheap way to achieve this goal. The economic impact of these treatments, therefore, is still sustainable considering the efficiency of natural mating during periods of increasing daylight length. In our study, both treatments for the synchronization of ovulation in both seasons provided good pregnancy rates, although P_4_-based treatment has reported a superior efficiency during the high season following the first AI, and during the low season when all AIs were cumulatively considered. The results derived from this study may give indications on how to improve reproductive efficiency in primiparous buffaloes in commercially managed buffalo herds.

## Figures and Tables

**Table 1:** In rows, descriptive statistics relative to the efficiency of the first AI recorded on primiparous buffaloes synchronized by Ovsynch (OV) or Progesterone based treatment (P_4_), and in columns, undergoing AI during periods characterized by Increasing Daylight Hours (IDH) and Decreasing Daylight Hours (DDH).

Treatment	% IDH (n)	% DDH (n)	% TOTAL (n)
**OV**	50.3 (146/290)	50.4 ^A^ (123/244)	50.4 ^A^ (269/534)
**P_4_**	57.4 ^x^ (147/256)	67.7 ^yB^ (149/220)	62.2 ^B^ (296/476)
**Pooled**	53.7 (293/546)	58.6 (272/464)	55.9 (565/1010)

Values in the same columns with different superscripts are significantly different (^A,B^, *p* < 0.01). Values in the same rows with different superscripts are significantly different (^x,y^, *p* < 0.05).

**Table 2:** Odds ratio for the logistic regression modeling the success at first AI. Third column reports the sign of the coefficient and fourth column the *p*-value. The covariates are in the first column.

	Odds Ratio	Coefficient	*p* Value
**(Intercept)**	0.311	negative	0.037
**treatment**	1.172	positive	0.365
**season DDH**	0.733	negative	0.096
**dip**	1.012	positive	1.265
**age calving**	1.000	positive	0.607
**treatm (P_4_) X season (DDH)**	2.233	positive	0.003

**Table 3:** Descriptive statistics on the efficiency of re-synchronization and reported pregnancies, over season (column) and re-synchronization protocols (rows) in primiparous buffaloes synchronized by Ovsynch (OV) or Progesterone based treatment (P_4_) and that underwent AI during periods characterized by Increasing Daylight Hours (IDH) and Decreasing Daylight Hours (DDH). Last column and last row report the pooled seasons and the pooled treatments, respectively.

Treatment	% IDH (n)	% DDH (n)	% TOTAL (n)
**OV**	72.0 ^A^ (188/261)	71.8 (204/284)	71.9 (392/545)
**P_4_**	82.2 ^B^ (166/202)	76.0 (218/287)	78.5 (384/489)
**Pooled**	76.5 (354/463)	73.9 (422/571)	75.0 (776/1034)

Values in the same columns with different superscripts are significantly different (^A,B^, *p* < 0.01).

## Data Availability

Data presented in this study are available on request from the corresponding author.
